# The expanding pattern of *Aedes aegypti* in southern Yunnan, China: insights from microsatellite and mitochondrial DNA markers

**DOI:** 10.1186/s13071-019-3818-8

**Published:** 2019-11-27

**Authors:** Pengbo Liu, Liang Lu, Jinyong Jiang, Yuhong Guo, Mingdong Yang, Qiyong Liu

**Affiliations:** 1 State Key Laboratory of Infectious Disease Prevention and Control, National Institute for Communicable Disease Control and Prevention, Chinese Center for Disease Control and Prevention, Beijing, 102206 China; 2 Yunnan Institute of Parasitic Diseases, Pu’er, 665000 China

**Keywords:** *Aedes aegypti*, Population genetics, Microsatellite loci, Expansion, Southwestern China

## Abstract

**Background:**

*Aedes aegypti*, the vector of dengue fever, was first reported in Yunnan in 2002. Now, this species is found in nine counties in border areas of south-west Yunnan. Related dengue fever outbreaks have been reported since 2013. The population genetics of *Ae. aegypti* in these areas were studied to explain the expansion history of this species.

**Methods:**

Fifteen natural populations of *Ae. aegypti* were sampled from six counties of Yunnan, and two laboratory populations from Guangdong and Hainan were also included in this study. A total of 12 microsatellite loci and three mitochondrial genes were analysed.

**Results:**

The results indicate that *Ae. aegypti* populations from Yunnan show similar genetic diversity. The 17 populations could be divided into three groups: the first group included populations from Longchuan, Ruili and Gengma, which are located in the southwest of Yunnan; the second group included populations from Jinghong and Menghai, in the south of Yunnan; and the third group included populations from Mengla and the two laboratory populations from Guangdong and Hainan. Both microsatellite and mtDNA data revealed that the genetic relationships of the populations corresponded to their geographic relationships.

**Conclusions:**

The results suggested that the expansion of *Ae. aegypti* from northern Myanmar and Laos to southern and southwestern Yunnan was a natural process. The effect of human activity on expansion was not obvious. Surveillance efforts should still be focused on border areas where *Ae. aegypti* does not occur, and a powerful control strategy should be applied to prevent outbreaks of dengue fever.

## Background

Dengue fever (DF) is the most rapidly spreading arboviral disease in the world, with *Aedes aegypti* representing the most important vector [1]. In 2009, the World Health Organization (WHO) reported that there were approximately 50 million new cases of dengue per year, and approximately 2.5 billion people are at risk of contracting DF around the world [2]. Bhatt et al. [3] estimated in 2013 that there were approximately 390 million dengue infections per year, and approximately 40% of the world population is at risk of infection from dengue. The latest WHO information indicated that the incidence of DF had increased 30-fold over the last 50 years and up to 50–100 million infections were estimated to occur annually in over 100 endemic countries, putting almost half of the world’s population at risk [4].

Before 2000, the distribution of *Ae. aegypti* in China was recorded in areas below 22° north latitude, including Hainan, Guangxi, Guangdong and Taiwan, and no *Ae. aegypti* were recorded in Yunnan [5]. This species was first found at the Jiegao Port of Ruili, a county south-west of Yunnan, in 2002 [6]. In 2006, *Ae. aegypti* was found in the county city of Ruili, which is approximately 5 km away from Jiegao Port. In 2008, and 2009, *Ae. aegypti* was found at the Guanlei wharf on the Mekong River and in the Mohan Port of Mengla County, respectively. In 2011, *Ae. aegypti* was found in Jinghong, Xishuangbanna. There were records of *Ae. aegypti* in Ruili, Mangshi, Mengla, Menghai, Jinghong, Yingjiang, Longchuan and Gengma counties by 2014 [7]. Dengue outbreaks have since been reported almost every year in Ruili, Jinghong, Mengla and Gengma in Yunnan [8, 9].

Yunnan is located in southwestern China in areas contiguous with Myanmar, Laos, and Vietnam and connected to Cambodia and Thailand *via* the Mekong River. These countries of Southeast Asia are all threatened by dengue fever transmitted by *Ae. aegypti* [10]. Most imported dengue fever cases in China arise from infections contracted in Southeast Asia [11]. Human travel and trade between Southeast Asia and China might not only export dengue fever cases to China but also export *Ae. aegypti* populations to border areas of Yunnan. However, the real reasons for the appearance of *Ae. aegypti* populations in Yunnan should be examined to develop an effective survey and control strategy for *Ae. aegypti* in related areas. There are two possible scenarios for the invasion of *Ae*. *aegypti* in Yunnan, China: (i) the mosquito expanded to Yunnan naturally, and populations from different areas are genetically independent; and (ii) the mosquito invaded Yunnan due to increasing transportation among countries, and populations from different areas are therefore linked by the highway system between countries and counties of Yunnan.

Because of the high variability, codominant expression, and broad genome distribution of microsatellites, they have been widely used as classic genetic markers in population genetic studies, providing information on population structure, patterns of gene flow, histories of introduction and colonization, or pathways of the expansion of invading species in new areas [12, 13]. There have been several reports of the successful development and use of microsatellite markers in *Ae. aegypti* from South America and Southeast Asia and on a global scale [14–17]. However, almost half of the microsatellite sequences of *Ae. aegypti* are located directly within or closely linked to repetitive elements, which could reduce the possibility of using these microsatellite sequences in genetic studies [16]. Therefore, in the present study, we isolated new microsatellites and validated their application in genetic research in *Ae. aegypti* populations of China.

Mitochondrial DNA (mtDNA) replicates independently from nuclear genomes and is materially inherited without recombination, so it can allow sharper genetic differentiation than nuclear DNA [18, 19]. The mitochondrial genes cytochrome *c* oxidase subunit 1 (*cox*1), NADH dehydrogenase subunit 4 (*nad*4), and NADH dehydrogenase subunit 5 (*nad*5) are frequently used to infer the phylogenetic relationships of mosquitoes. *cox*1 is the most conserved of the three cytochrome oxidases encoded by mtDNA [20] and shows greater sequence variation at the inter-species level than at the intra-species level [21]. The *nad*4 and *nad*5 loci are highly polymorphic and have often been used to conduct genetic analysis of *Ae. aegypti* [22–24].

Based on population genetics analysis with microsatellite and mtDNA loci, we sought to study two issues in the *Ae. aegypti* population in Yunnan, China: (i) the scenario of *Ae. aegypti* invasion of Yunnan, to develop an effective strategy for the surveillance and control of the *Ae. aegypti* population; and (ii) considering the expansion of *Ae. aegypti*, the possibility that this species will continue to spread within and outside of Yunnan. Molecular markers that can be used for population identification are needed to distinguish *Ae. aegypti* populations from Yunnan, Guangdong and Hainan.

## Methods

### Mosquito samples

**Fig. 1:**
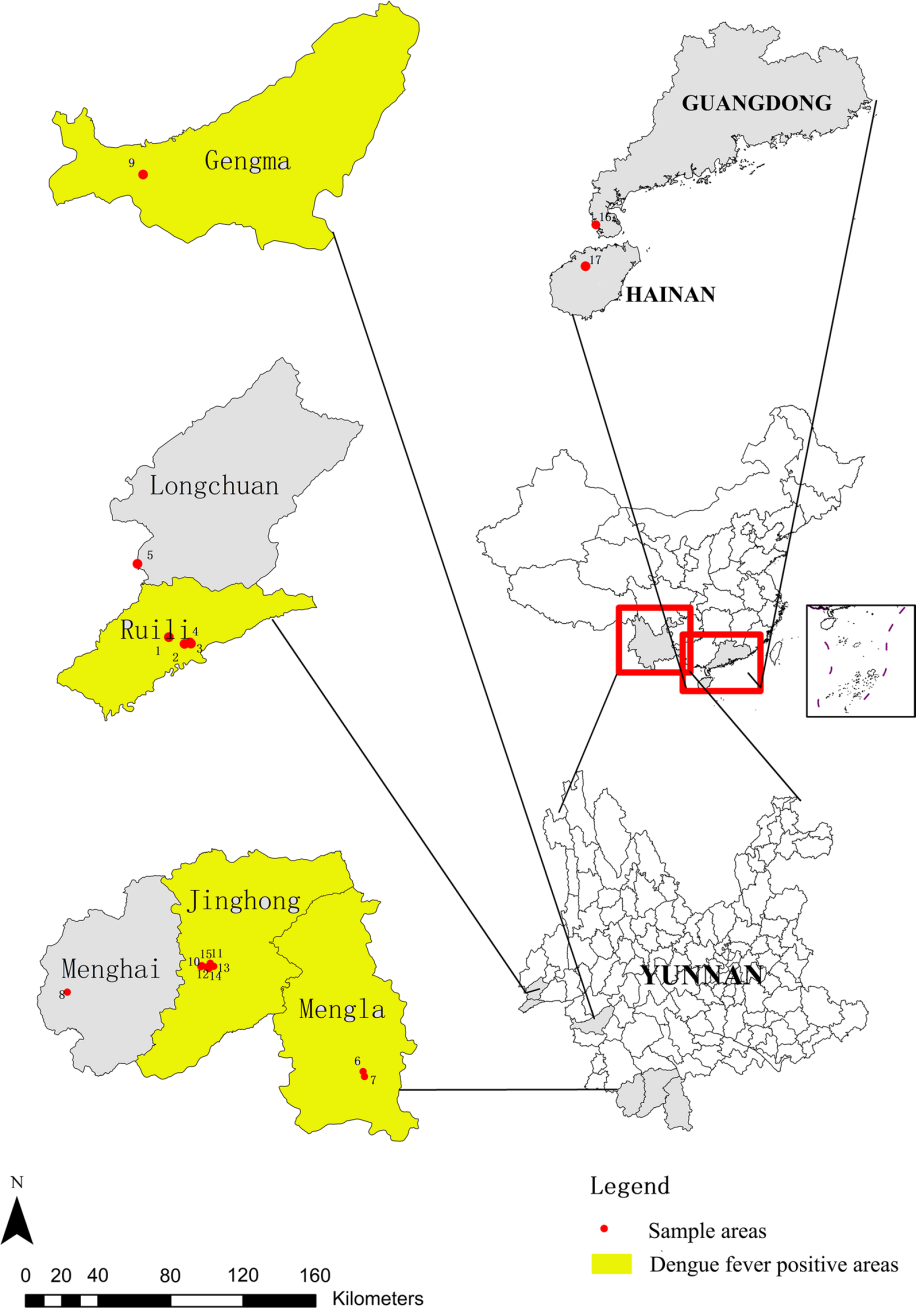
Sampling locations in Yunan (*n *= 15), Guangdong (*n *= 1) and Hainan (*n *= 1). Specifically, in Yunan, there was 1 sampling site in Longchuan, 4 in Ruili, 1 in Gengma, 1 in Menghai, 6 in Jinghong and 2 in Mengla county. In 2016, *Ae. aegypti* was reported from these counties. Among these counties, Ruili, Gengma, Jinghong and Mengla were dengue fever-positive areas and are labelled in yellow

Mosquitoes from fifteen sites in Yunnan were sampled in the autumn of 2016 based on the distribution of *Ae. aegypti*: four from Ruili, one from Longchuan, two from Mengla, one from Menghai, one from Gengma, and six from Jinghong (Fig. 1, Table 1). Adult mosquitoes were collected using a sweep net from each sampling area, and the larvae and pupae were collected with ovitraps. Each sampling site was located with GPS. Samples of 2 laboratory populations from Guangdong and Hainan, which were collected from the distribution areas in the two provinces, were also used in this research (Fig. 1, Table 1). Samples identified as *Ae. aegypti* were preserved in 95% ethanol in labelled vials and stored at 4 °C before the isolation of genomic DNA.

**Table 1:** Sampling information for the *Ae. aegypti* populations used in the present study

Population code (collection site)	Region	*n*	Geographical coordinates
MML (Mengmao Road)	Ruili	47	24°00′57″N, 97°50′39″E
NAM/Nanmen	Ruili	35	24°01′52″N, 97°48′18″E
RJL/Ruijin Road	Ruili	32	24°01′09″N, 97°51′27″E
JGL/Jiegang Road	Ruili	47	24°00′59″N, 97°51′35″E
LYC/Laying village	Longchuan	32	24°11′50″N, 97°43′38″E
GEM/Mengding	Gengma	24	22°00′21″N, 99°04′58″E
LBH/Department store	Mengla	28	21°27′44″N, 101°34′05″E
JGC/Old Factory	Mengla	27	21°29′13″N, 101°33′38″E
DAL/Daluo	Menghai	47	21°42′37″N, 100°12′23″E
HHY/Huahuijiayuan	Jinghong	32	22°00′21″N, 100°47′03″E
NKK/Nakunkang	Jinghong	32	21°59′52″N, 100°47′47″E
GAZ/Gaozhuang	Jinghong	32	22°00′33″N, 100°49′13″E
FTC/Fantaichang	Jinghong	32	22°01′27″N, 100°48′11″E
ZYY/Zhouyiyuan	Jinghong	32	22°00′46″N, 100°45′39″E
CAJ/Cangjiangzhizaochang	Jinghong	32	22°00′25″N, 100°45′24″E
LGD/Wushi	Guangdong	32	20°33′19″N, 109°50′50″E
LHN/Danzhou	Hainan	32	19°31′16″N, 109°34′51″E

*Abbreviation*: *n*, number of mosquitoes

### DNA extraction

Total nucleic acids were extracted from each individual of *Ae. aegypti* with a magnetic bead-based semi-automatic system (Biotake, Beijing, China) following the manufacturer’s protocol. The DNA samples were stored at − 20 °C until further analysis.

### Microsatellite analysis

Twelve polymorphic microsatellite loci were used in this study: 11 pairs of primers were obtained *via* a genome-based method [16], and 1 pair of primers was selected from reported data [25]. The primer sequences and information are summarized in Table 2. The forward sequence of each primer was end-labelled with one of three different fluorescent dyes (FAM, ROX and HEX). Each amplification reaction was performed in a CFX-96 PCR Amplification instrument in a final volume of 25 μl (12.5 μl of 2× PCR mix, each primer at 1 μM, 1.5 μl of DNA, and 9 μl of dd H_2_O). The PCR conditions were as follows: 95 °C for 10 min, followed by 30 cycles at 95 °C for 1 min, a different annealing temperature for each locus for 30 s, and 72 °C for 45 s. A final extension step was conducted at 72 °C for 10 min. The PCR product standard mixture was analysed on the ABI3730XL Genetic Analyzer (Applied Biosystems, Foster City, USA), and the data analysed using GeneMapper software.

**Table 2:** Characteristics of the 12 microsatellite loci of *Ae. aegypti* used in the present study

Locus	Repeat motif	Primer sequence (5′–3′)	Annealing T (°C)	Allele size (bp)	GenBank ID
AC10	AC	F: CAATTATGATCCGTGGTGTT	49	185	MK733227
		R: GCGAAGAATGGTGGTCTA			
1209	AC	F: GCAATCTGGTCGTCGTTA	54	370	MK733223
		R: GGCTATATCTGATCTGGTGAT			
AG3	AG	F: GACTAAGCAGGACGACAG	52	323	MK733228
		R: AAGCAGGTTGATGAGATTCT			
TTC5	TTC	F: ACATTTGTTTTGCTATTGTGG	55	154	MK733232
		R: AAGAACATTATGCTAAAAAGCAG			
AAG6	AAG	F: AGGATCTTTTCGTAAGAAGCA	55	150	MK733224
		R: GGAATTGTTCTCTACATGCTG			
AAT1	AAT	F: AAGGAACACTAGTTCGGTAGG	55	155	MK733225
		R: GAGCTGTTCAAGAACACAAGT			
TTGT	TTGT	F: TGTTTGAGCTGAAAATCTCAT	55	127	MK733233
		R: GTCAAATCGGAGGTAGTGAAT			
AC1	AC	F: GAGTATATCGGCCTCCAATAC R: CATACAGGTACACGCTAGGAT	55	146	MK733226
CGA	CGA	F: TGCAGTTCTACAACTCCTTTT	55	156	MK733229
		R: TTACCAGTTGAAGTTGATTGC			
GAT	GAT	F: CGTATCGTGTTACGCTATCTC	55	154	MK733230
		R: GTAGGCAAAACGATCACAGT			
TC	TC	F: TTCATCTTTCACTCATTCCAC	55	165	MK733231
		R: CAAGTGCCCTATAGTGTTTGT			
AG5	AG	F: TGATCTTGAGAAGGCATCCA	55	170	^a^
		R: CGTTATCCTTTCATCACTTGTTTG			

^a^Published by Slotman et al. [25]

*Abbreviation*: T, temperature

The genetic diversity of every locus was characterized by estimating the number of different alleles (*N*_*a*_), number of effective alleles (*N*_*e*_), Shannonʼs information index (*I*), observed heterozygosity (*H*_*o*_), expected heterozygosity (*H*_*e*_) and positive inbreeding coefficient (*F*_*IS*_) with GenAIEx (version 6.501) [26]. The value of *Nm* was calculated with the formula 1/4(*F*_*ST*_-1). PIC-Calc 0.6 [27] was used to assess polymorphic information content (PIC) across all 12 loci. Linkage disequilibrium (LD) between pairs of locations and deviations from Hardy–Weinberg equilibrium (HWE) were assessed, and analysis of molecular variance (AMOVA) was performed with Arlequin software (version 3.5.2.2) [28]. Fstat (version 2.9.3) [29] was used to compute the estimated allelic richness (r) in each population. Microchecker software (version 2.2.3) [30] was used to check the presence and frequencies of null alleles at each locus.

Two approaches were used for testing genetic bottlenecks at the microsatellite loci for each population: in the first, the mean ratio of the number of alleles to the range of allele size (M ratios) was calculated to infer possible genetic bottlenecks in the more distant past [31] in Arlequin3.5 [32], and the second was performed using the infinite allele model (IAM) [33], stepwise mutation model (SMM) [34], and two-phase model (TPM) [35], which were run twice for each population, assuming that the percentage of stepwise mutations was 80%. The statistical significance of the tests was assessed for each population across all loci with the Wilcoxon signed-rank test available in Bottleneck [36].

Genetic distance was calculated using GenAIEx (version 6.501) [26], and the result was used to conduct principal components analysis (PCoA) based on the codom-genotypic genetic distance. A neighbour-joining (NJ) tree was generated with Mega v.6.0 [37] based on genetic distance. Factorial correspondence analysis (FCA) was accomplished with Genetix (version 4.05) [38]. The population genetic structure was determined by a Bayesian clustering method using STRUCTURE software (version 2.3.4) [39]. A model in which allele frequencies correlated within populations was assumed (λ was set at 1, the default value). The software was run with the option of admixture, allowing for some mixed ancestry within individuals, and α was allowed to vary. Twenty independent runs were conducted for each value of *Κ* (*K *= 1 to 10), with a ‘burn-in’ period of 50,000 iterations and 250,000 replications. The method of Evanno et al. [40] was used to determine the most likely number of clusters. The results of 20 replicate runs for each value of *K* were combined using the Greedy algorithm of Clumpp 1.1.1 [41], and summary outputs for each value of *K* were displayed graphically using Distruct v1.1 [42]. Isolation by distance (IBD) was estimated with Mantel’s test employing the IBD Web Service using the correlation between genetic and geographic distances *via* regression of pairwise *F*_*ST*_/(1 − *F*_*ST*_) on the natural logarithm (*Ln*) of straight-line geographical distance [26].

### Mitochondrial DNA analysis

Three mitochondrial genes, *cox*1, *nad*4 and *nad*5, were used to explore the polymorphism of all samples. The three fragments were amplified and sequenced with previously reported primers [18, 43]. The haplotypes were numbered according to their order frequency. Basic sequence statistics, including nucleotide diversity (π) and the neutrality tests of Tajima and Fu, were computed with DnaSP V5 [44]. Phylogenetic networks based on the *cox*1, *nad*4 and *nad*5 sequences were constructed with the Network program (version 5.0) [45]. Similarly, IBD was estimated with Mantel’s test separately for these three mitochondrial genes using the correlation between genetic and geographic distances in GenAIEx (version 6.501).

## Results

### Quality of microsatellite markers

A total of 585 individual mosquitoes collected from 17 populations were successfully genotyped at all 12 microsatellite loci (Additional file 1: Table S1). All loci were polymorphic, and the number of alleles ranged from 6 to 16. The average number of alleles per locus ranged from 5.98 (GAT) to 15.99 (TC). The PIC of all loci ranged from 0.199 to 0.775, and 7 of them showed PIC values greater than 0.5 (Table 3). The minimum mean number of alleles of all loci was recorded for the FTC population from Jinghong (3.083) and the maximum was recorded for the CAJ population from Jinghong (6.333). The minimum *H*_*o*_ was found in GEM (0.309), and the maximum *H*_*o*_ was found in LYC (0.464) (Table 4). The minimum *H*_*e*_ was observed in LBH (0.447), and the maximum *H*_*e*_ was observed in CAJ (0.630) (Table 4). The same situation occurred for *N*_*e*_ and I: the minimum value was found in NKK (*N*_*e*_= 2.031, *I *= 0.817), the maximum value was found in CAJ (*N*_*e*_= 3.107, *I *= 1.283) (Table 4).

**Table 3:** Polymorphic information of the 12 microsatellite loci of *Ae. aegypti* from Yunnan Province, China

Locus	No. of individuals	No. of alleles	Richness	PIC
AC10	585	15	14.96	0.722
1209	585	13	13.00	0.707
AG3	585	12	12.00	0.707
TTC5	585	10	9.92	0.661
AAG6	585	15	14.96	0.651
AAT1	585	10	9.98	0.199
TTGT	585	15	15.00	0.775
AC1	585	12	11.94	0.426
CGA	585	7	6.98	0.409
GAT	585	6	5.98	0.264
TC	585	16	15.99	0.631
AG5	585	6	6.00	0.459
Mean		11.42	11.40	0.551

**Table 4:** Genetic variation in 17 populations of *Ae. aegypti* from Yunnan Province, China, averaged over 12 microsatellite loci

Pop	*N*	*N*_*a*_	*N*_*e*_	*I*	*H*_*o*_	*H*_*e*_	*uH*_*e*_	*F*_*IS*_	IAM	TPM	SMM
MML	46.583	5.583	2.505	1.029	0.407	0.519	0.525	0.280	0.564	0.419	**0.003**
NAM	35.000	4.167	2.507	0.957	0.421	0.507	0.514	0.165	0.113	0.117	0.102
RJL	32.000	4.333	2.366	0.929	0.378	0.492	0.500	0.245	0.280	0.478	**0.026**
JGL	46.750	4.667	2.182	0.856	0.417	0.455	0.460	0.140	0.390	0.585	**0.008**
LYC	31.833	4.917	2.623	1.051	0.464	0.547	0.556	0.137	0.337	0.595	0.185
LBH	27.917	3.750	2.237	0.834	0.353	0.447	0.455	0.145	0.363	0.367	0.318
JGC	27.000	3.833	2.412	0.852	0.346	0.452	0.461	0.217	0.175	0.184	0.305
DAL	46.500	4.750	2.612	1.033	0.428	0.550	0.556	0.197	0.146	0.350	0.197
GEM	22.250	5.250	2.854	1.135	0.309	0.580	0.594	0.484	0.169	0.179	0.059
HHY	31.917	3.333	2.324	0.843	0.434	0.477	0.485	0.103	**0.045**	0.056	0.470
NKK	32.000	3.750	2.031	0.817	0.378	0.470	0.478	0.231	0.472	0.501	0.210
GAZ	31.500	3.833	2.288	0.922	0.422	0.519	0.528	0.254	0.249	0.266	0.432
FTC	31.583	3.083	2.170	0.796	0.436	0.461	0.469	0.039	**0.007**	**0.047**	0.443
ZYY	31.917	3.417	2.304	0.863	0.393	0.489	0.496	0.153	**0.007**	**0.049**	0.423
CAJ	31.250	6.333	3.107	1.283	0.335	0.630	0.640	0.456	0.610	0.385	**0.003**
LGD	31.667	3.500	2.180	0.829	0.328	0.457	0.464	0.300			
LHN	31.500	3.417	2.271	0.809	0.361	0.448	0.455	0.167			
Mean	33.480	4.230	2.410	0.932	0.389	0.500	0.508	0.222			

*Notes*: *P*-values for genetic bottleneck detection using the Wilcoxon signed-rank test under the infinite allele (IAM), step-wise mutation (SMM) and two-phase mutation (TPM) models. Bold characters denote a significant heterozygote deficiency (*P* < 0.05) after correction for multiple testing by the sequential Bonferroni procedure

*Abbreviations*: *N*, effective number of samples; *N*_*a*_, number of alleles per population; *N*_*e*_, effective allele; *I*, Shannonʼs information index; *H*_*o*_, observed heterozygosity; *H*_*e*_, expected heterozygosity; *uH*_*e*_, unbiased expected heterozygosity; *F*_*IS*_, inbreeding coefficient

### Microsatellite analysis

#### Genetic diversity

The highest *Ho* was found in the LYC population, while the lowest *Ho* was found in the GEM population, with an average value of 0.389. The *He* values were higher than the *Ho* values at all sites. The HWE test suggested that all populations from Yunnan were in HWE (Additional file 2: Table S2). Similarly, 207 out of 1122 tests for linkage disequilibrium were significant after correction, while no consistency was found at any loci across all populations. The M-ratios of five populations (HHY and NKK of Jinhong, JGC of Mengla, JGL of Ruili, LYC of Longchuan) were low and indicated historical bottlenecks in these populations. The bottleneck test between the TPM and SMM models showed that there were 6 populations from Ruili and Jinghong (MML, RJL and JGL of Ruili, FTC, ZYY and CAJ of Jinhong) that had experienced recent population extinction (Table 4).

#### Genetic structure

**Fig. 2:**
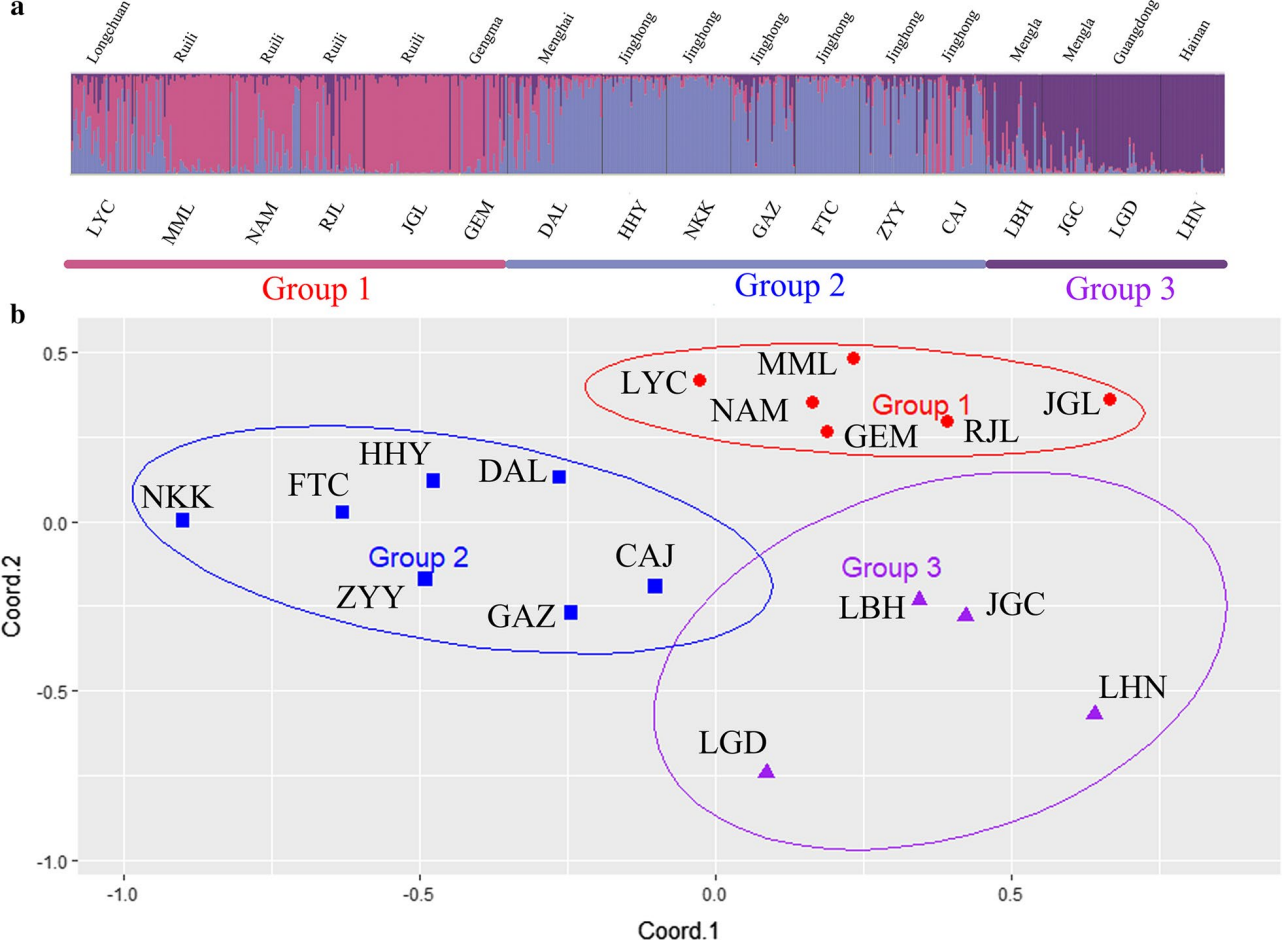
Genetic structure within 17 populations of *Ae. aegypti*. **a** STRUCTURE bar plots indicate the relatedness of *Ae. aegypti* populations based on 12 microsatellite loci. Each vertical bar represents an individual. The height of each bar indicates the probability of assignment to each of *K* optimal clusters (different colours) determined using the Δ*K* method of Evanno et al. [40]: *K *= 3. **b** Genetic similarities among populations of *Ae. aegypti* based on principal coordinates analyses (PCoA); the oval shapes indicate the 85% confidence interval

**Fig. 3:**
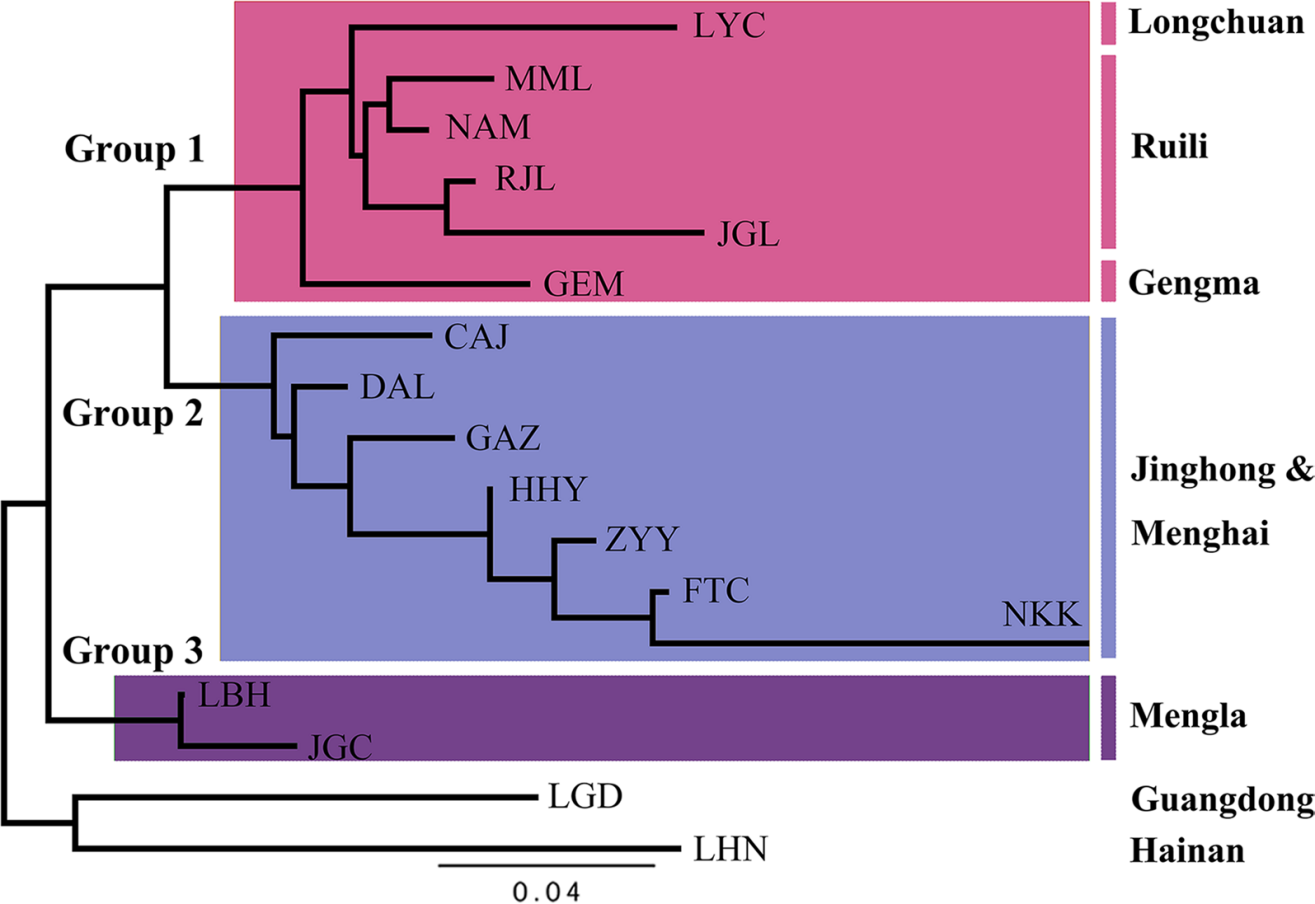
UPGMA cluster analysis based on the genetic distance of all populations, showing the relationships between sampled cities. The red, blue and purple indicate Group 1, Group 2 and Group 3, respectively

Bayesian clustering analysis with STRUCTURE in these populations identified the most likely number of clusters as 3: the samples from Ruili, Longchuan and Gengma were in the first cluster (Group 1); the samples from Jinghong and Menghai made up the second cluster (Group 2); and the populations from Mengla, Guangdong and Hainan belonged to the third group, which showed a close relationship among these populations (Group 3) (Fig. 2a). The map of PCoA (Fig. 2b) and the UPGMA tree (Fig. 3) showed similar results, indicating 3 groups, as indicated above.

#### Genetic differences

**Fig. 4:**
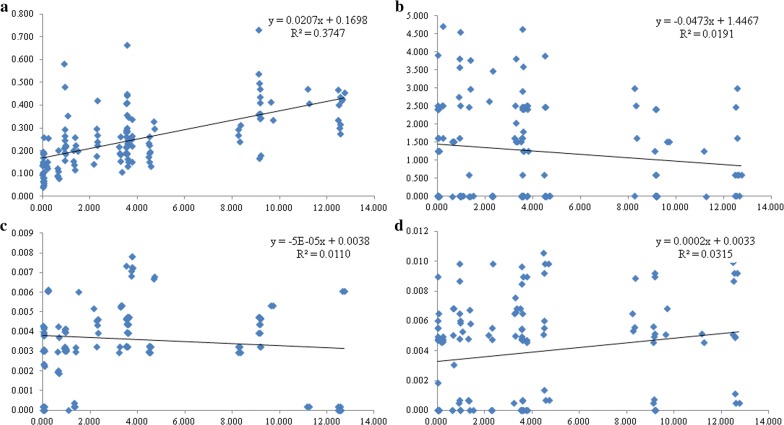
Analysis of the relationship between genetic distance [*F*_*ST*_/(1 − *F*_*ST*_)] and geographical distance [ln(km)] based on 12 microsatellite loci (*R*^2^= 0.3747, *P* < 0.01) (**a**), *cox*1 (*R*^2^= 0.011, *P* < 0.05) (**b**), *nad*4 (*R*^2^= 0.0191, *P* < 0.140) (**c**), and *nad*5 (*R*^2^= 0.0315, *P* < 0.870) (**d**)

The *F*_*IS*_ values based on 12 loci were positive across all populations (Additional file 2: Table S3), which indicated different degrees of inbreeding within populations. The maximum *F*_*IS*_ value was found in GEM (0.486), and the minimum *F*_*IS*_ value was found in FTC (0.071). The *F*_*ST*_ and *Nm* values are shown in Additional file 2: Table S4. The lowest pairwise *F*_*ST*_ value among the 17 populations was found between MML and NAM (*F*_*ST*_= 0.017), and these two populations were both from Ruili city. The pairwise *F*_*ST*_ values were always lower between populations from the same city than between those from different cities. The *F*_*ST*_ values between LGD, LHN and other populations were especially high compared to those for other populations from Yunnan. The value of *N*_*m*_ was between 0.807 (LHN and NKK) and 14.339 (MML and NAM), and most of the *N*_*m*_ values among the populations were greater than 1, indicating that there was a certain degree of communication among these populations of *Ae. aegypti*. According to the AMOVA results, the percentage of variation between groups was only 8.71%, while the percentage of variation within individuals was 63.48%, and the *F*_*IT*_ (total inbreeding coefficient) value was 0.3652. The results of the Mantel test showed a significant positive correlation between Nei’s standard genetic distance and geographical distance (*R*^2^= 0.3747, *P* < 0.01), as shown in Fig. 4a.

### DNA sequencing analysis

There were 549 mtDNA *cox*1 (507 bp), 544 mtDNA *nad*4 (553 bp) and 534 mtDNA *nad*5 (414 bp) nucleotide sequences obtained from the 17 populations.

The mitochondrial DNA results showed that the *cox*1 gene had 11 haplotypes. The haplotype diversity index of the total population was 0.5670, and the nucleotide diversity index was 0.0034. The average number of nucleotide differences (*k*) was 1.687. The LYC population harboured the most haplotypes (*n *= 4): Hap1, Hap9, Hap10 and Hap11. There were 5 populations (LGD, LHN, MML, NAM and RJL) exhibiting only one type of haplotype. The *nad*4 gene had 15 haplotypes. The haplotype diversity index of the total population was 0.5603, and the nucleotide diversity index was 0.0224. The MML population from Ruili and the DAL population from Menghai exhibited the most haplotypes (*n *= 5). Four of the 5 populations with only one haplotype of *nad*4 were the same as those with only one *cox*1 haplotype; these populations were LGD, LHN, NAM, RJL and HHY. The *nad*5 gene presented 10 haplotypes, and Hap1 was detected in all geographical populations. The indices of the total population were 0.503 for haplotype diversity, 0.005 for nucleotide diversity and 1.920 for the average number of nucleotide differences.

**Fig. 5:**
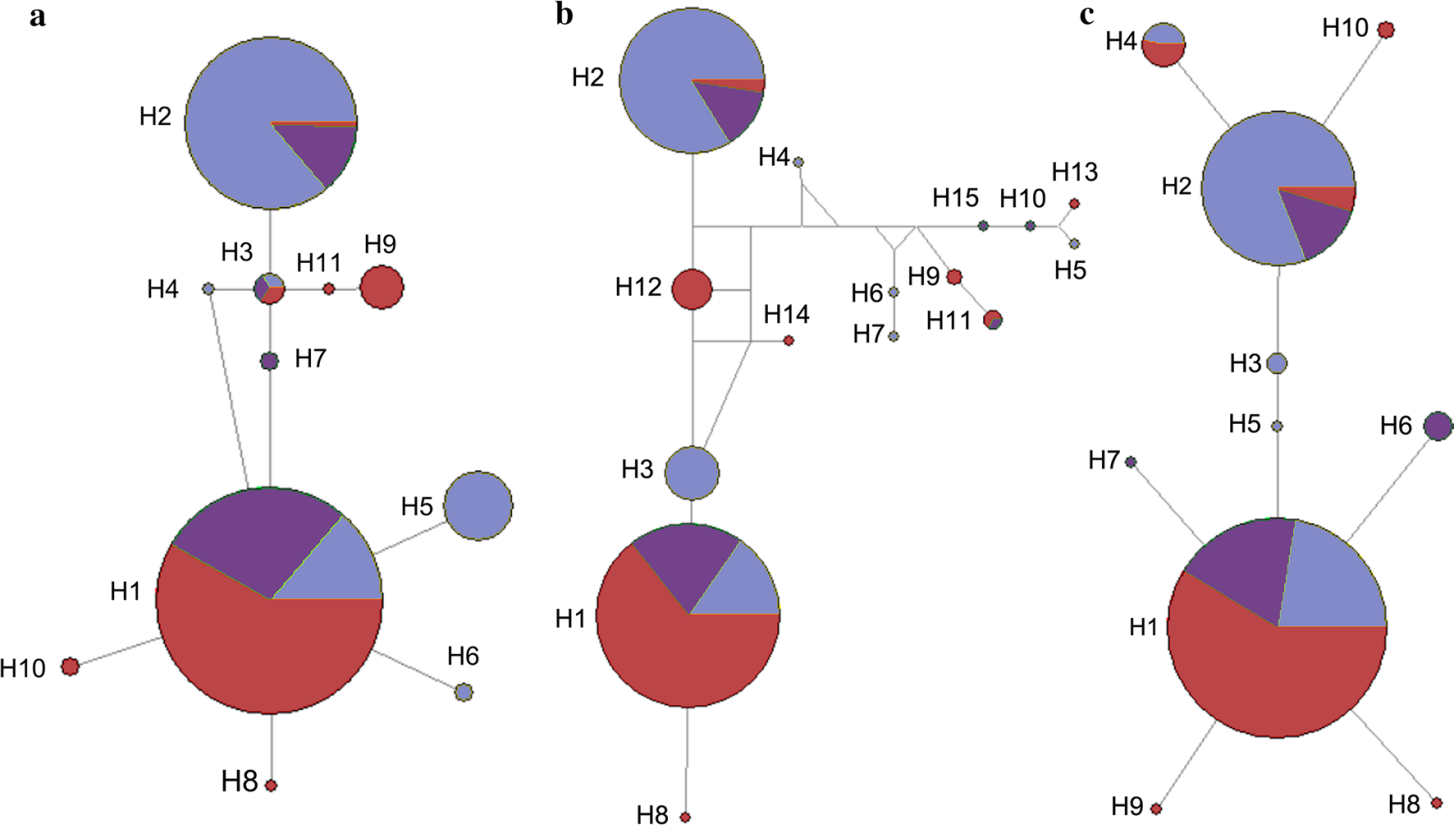
Genealogical relationships of *Ae. aegypti* based on the median-joining haplotype network of 3 mitochondrial fragments: *cox*1 (**a**); *nad*4 (**b**) and *nad*5 (**c**). Each line in the network represents a single nucleotide mutation. The size of the nodes corresponds to the frequency of the haplotypes. Colours indicate the collection regions: Group 1 (red), Group 2 (blue), and Group 3 (purple)

The results of the population expansion test showed that the *P*-values with negative test values were all less than 0.05, indicating that these populations had not expanded during their history. The haplotype network maps (Fig. 5) showed that each mitochondrial gene exhibited two dominant haplotypes. One was found in a large number of individuals from Jinghong and Menghai, and the other was found in many individuals collected in Ruili, Longchuan and Gengma. Most of the other haplotypes differed from these two haplotypes. No correlation was detected between genetic and geographic distances for all three mtDNA genes (*cox*1: *R*^2^= 0.011, *P* < 0.05; *nad*4: *R*^2^= 0.019, *P* < 0.140: *nad*5: *R*^2^= 0.031, *P* < 0.870), as shown in Fig. 4b–d.

## Discussion

*Aedes aegypti* is a mosquito species distributed worldwide in tropical and subtropical regions. No *Ae. aegypti* were found in any survey conducted before 2000 in Yunnan, and *Ae. aegypti* larvae were captured for the first time at the Jiegao Port of Ruili in 2002. The larvae of *Ae. aegypti* are mainly found in jars and flowerpots indoors and outdoors. Female mosquitoes usually suck blood near their breeding grounds. The flying distance of adults is usually short, and the average diffusion radius is within 100 m [46]. *Aedes aegypti* does not enter diapause in low temperatures and short photoperiods [47] and therefore cannot expand over long distances *via* diapause eggs, which is the main reason that *Ae. albopictus* expanded from Asia to North America and Europe [48, 49]. The mode of *Ae. aegypti* expansion in border areas of China and Southeast Asian countries could be revealed *via* population genetics research with microsatellite and mtDNA markers.

### Genetic diversity and demographic history

Genetic diversity is one of the most important attributes of populations. The decline in genetic diversity among geographical populations could be an indicator of the bottleneck effect or founder effect that occurred in the history of populations expanding or invasion [50]. According to the results of this study, the genetic diversities of the different populations are similar, with *H*_*o*_ values ranging from 0.309 to 0.467, with an average value of 0.389. All the natural populations conformed to Hardy-Weinberg equilibrium. These results indicated that there was no obvious decline in genetic diversity among these populations, which indicated that the *Ae. aegypti* populations from these areas were not the offspring of one or two invasion events from Southeast Asian countries but the result of successive expansions *via* different routes.

The results of the bottleneck tests (Table 4) showed two scenarios of the demographic history of *Ae. aegypti* of Yunnan. The M-ratio test indicated that five populations had experienced slight bottlenecks in their history because the M-value of these populations was just below the critical value of 0.68 proposed by Garza & Williamson [31] and that the other populations had not suffered bottlenecks in their distant past. The results of Wilcoxon signed-rank tests showed that only some populations from Ruili and Jinghong had experienced bottlenecks recently. The first scenario meant that *Ae. aegypti* expanded naturally from Southeast Asia to Yunnan, rather than being imported with human activities, and the second indicated that only some *Ae. aegypti* populations in urban areas have been under pressure from mosquito control measures applied during dengue fever outbreaks in recent years.

### Genetic structure and differentiation

The results of population clustering were consistent with the geographical distribution of the populations. Longchuan, Ruili and Gengma are located in the southwestern part of Yunnan, contiguous with Myanmar. Populations from these areas were clustered in group 1 (Figs. 2, 3). The second group was composed of populations from Jinghong and Menghai, which are contiguous with northeastern Myanmar. The third group included two populations from Mengla, which is the only county of Xishuangbanna bordering Laos. This result suggests that the populations from these areas exhibited at least three sources and that expansion had occurred independently from adjacent areas of Myanmar and Laos. According to the results of genetic structure and PCoA, two laboratory populations (LBG and LBH) were closely related to populations from Mengla. Among the six counties, only Mengla is adjacent to Laos, which could be the reason that populations from Mengla are separating from other populations. Because Laos is economically closely related to Vietnam, and Vietnam is geographically close to Hainan and Guangdong, the populations of *Ae. aegypti* from Guangdong and Hainan are relatively closely related to populations of Mengla.

The results of the IBD test based on microsatellite markers showed that there was a significant positive correlation between geographical distance and genetic distance (*R*^2^= 0.375) (Fig. 4a). This finding suggested that the expansion of *Ae. aegypti* in collection areas was a natural process, rather than a process caused by human activities. However, the IBD tests based on mtDNA markers did not show a positive correlation, which suggested that the mutation rate of the mtDNA markers was lower than that of microsatellite markers and that the expansion of *Ae. aegypti* in these areas was a recent event.

### The cause of *Ae. aegypti* expansion in southwestern Yunnan

Because of the close relationship with dengue fever, considerable attention has been paid to the occurrence of *Ae. aegypti* in Yunnan by public health authorities and academic institutes. Local CDC entities carry out specific surveillance in border areas of Yunnan [7]. Shi et al. [51] studied the population genetics of *Ae*. *aegypti* populations collected from six counties of Yunnan. Their results showed that all populations could be divided into three groups, two of which included populations from Jinghong and Ruili, respectively, and the third included populations from the other four counties (Menghai, Mengla, Gengma and Longchuan), which was different from the results of the present study. This relationship was not in accordance with the geographical relationship of populations, and could not be explained with any reasonable scenario of the expansion of *Ae. aegypti* in this area.

The occurrence of *Ae. aegypti* in high-latitude areas has not only been reported in southern Yunnan, China. Positive breeding sites of *Ae. aegypti* were noted at Darjeeling, West Bengal, India in 2006 [52]. The synchronous invasion of *Ae. aegypti* in northern South and Southeast Asia suggested that the northward expansion of this species was related to climate change on a large spatial scale. From 1954 to 2006, the annual mean temperature of Jinghong increased by approximately 1.3 °C, from 21.8 °C to 23.1 °C, and the increase was more obvious from the 1980s onward [53]. Therefore, the warmer climate of southwestern Yunnan should be the primary factor responsible for the expansion of *Ae. aegypti* in these areas. However, because this species does not enter diapause, its occurrence in other areas of Yunnan will be determined by the critical low temperature threshold for the survival of this species [54].

Hlaing et al. [55] studied the population genetics of *Ae. aegypti* populations from mainland Southeast Asia. The results suggested that human transportation routes have resulted in passive long-distance migration of *Ae*. *aegypti* in these areas (*R*^2^= 0.111, *P* < 0.05). However, the results of the IBD test in the present study (*R*^2^= 0.375, *P* < 0.01) indicated the role of human transportation in the expansion of *Ae*. *aegypti* in southwestern Yunnan was not as significant as in Southeast Asia. *Aedes aegypti* occurred independently in different areas in southwestern Yunnan from 2003 onward, indicating only a short history of these invasions. Because of the complex topography of Yunnan, transportation among these areas (Ruili, Gengma and Jinghong) is not convenient. Thus, the expansion of *Ae. aegypti* among these areas has not been found according to the results of the present study, and the distribution areas of the species were still restricted to border areas as of 2017 [56].

### Implications for vector surveillance

After the invasion of *Ae. aegypti* in Yunnan, dengue fever outbreaks were reported from four county areas of Yunnan (Ruili, Gengma, Jinghong and Mengla) [56]. Dengue fever became the most important vector-borne disease in Yunnan in 2013 [57]. Since the first report of *Ae. aegypti* in Yunnan, this species has only been found in border areas, although it was reported in eight county areas in 2017 [56]. There has been no evidence of expansion of distribution areas from border areas to inland areas of Yunnan. According to the results of the present study, *Ae. aegypti* invaded different areas of Yunnan independently, and there is no evidence of expansion among these areas. Therefore, the most important measures that can be adopted by the public health authority of Yunnan are surveillance and control of *Ae*. *aegypti* in border areas, especially in counties in areas that are currently negative for *Ae*. *aegypti*.

## Conclusions

The populations of *Ae. aegypti* in Yunnan can be divided into three groups according to population genetics. The genetic relationships of these populations were concurrent with their geographical relationships. This result suggested that the occurrence of *Ae. aegypti* in Yunnan is a result of the expansion of its distribution areas, rather than imported events closely related to human activity. Thus far, populations of *Ae. aegypti* have only occurred in border areas of Yunnan, and the species has not colonized inland counties of Yunnan. According to the topographic and climatic differences between border areas and inland areas of Yunnan, it should be difficult for the population of *Ae. aegypti* to expand to inland areas of Yunnan in a short time. Therefore, efforts aimed at the surveillance of *Ae. aegypti* and control of dengue fever should focus on the border areas of Yunnan, especially the counties and ports that are currently free of *Ae. aegypti*.

## Supplementary information

**Additional file 1: Table S1**. The microsatellite short tandem repeat data.

**Additional file 2: Table S2.** Results of Hardy-Weinberg equilibrium. **Table S3.** *F*_*IS*_ per population. **Table S4.** Population *F*_*ST*_ (lower left) and *N*_*m*_ (top right) matrix table.

## Data Availability

Data supporting the conclusions of this article are included within the article and its additional files. The microsatellite short tandem repeat dataset generated during this study are provided in Additional file 1: Table S1. The newly generated mitochondrial DNA sequences were submitted to the GenBank database under the accession numbers MK984840–MK985388 (*cox*1 gene); MK985923–MK986466 (*nad*4 gene); MK985389–MK985922 (*nad*5 gene).

## Abbreviations

AMOVA: analysis of molecular variance
*cox*1: cytochrome *c* oxidase subunit 1 gene
DF: dengue fever
FCA: factorial correspondence analysis
HWE: Hardy–Weinberg equilibrium
IAM: infinite allele model
IBD: isolation by distance
LD: linkage disequilibrium
mtDNA: mitochondrial DNA
*nad*4: NADH dehydrogenase subunit 4 gene
*nad*5: NADH dehydrogenase subunit 5 gene
NJ: neighbour-joining
PCoA: principal components analysis
PIC: polymorphic information content
SMM: stepwise mutation model
TPM: two-phase model
WHO: World Health Organization
