# Supplementary material for: The expanding pattern of Aedes aegypti in southern Yunnan, China: insights from microsatellite and mitochondrial DNA markers
Source: Parasit Vectors. 2019 Nov 27;12:561. doi: 10.1186/s13071-019-3818-8 (PMC6880496; doi:10.1186/s13071-019-3818-8)
Supplement: Supplementary file 2 — Additional file 2: Table S2. Results of Hardy-Weinberg equilibrium. Table S3. FIS per population. Table S4. Population FST (lower left) and Nm (top right) matrix table. [file 13071_2019_3818_MOESM2_ESM.docx]

**Additional file 2: Table S2.** Results of Hardy-Weinberg equilibrium

| Pop. | 1 | 2 | 3 | 4 | 5 | 6 | 7 | 8 | 9 | 10 | 11 | 12 | Mean |
| --- | --- | --- | --- | --- | --- | --- | --- | --- | --- | --- | --- | --- | --- |
| MML | 0.000 | 0.001 | 0.001 | 0.000 | 0.008 | 0.000 | 0.000 | 0.000 | 0.397 | 0.042 | 0.011 | 1.000 | 0.122 |
| NAM | 0.086 | 0.398 | 0.049 | 0.067 | 0.000 | 0.000 | 0.000 | 1.000 | 0.009 | 1.000 | 0.000 | 0.146 | 0.230 |
| RJL | 0.001 | 0.911 | 0.006 | 0.366 | 0.013 | 0.019 | 0.000 | 0.047 | 1.000 | 1.000 | 0.000 | 0.497 | 0.322 |
| JGL | 0.004 | 0.000 | 0.000 | 0.000 | 0.000 | 0.000 | 0.000 | - | 0.156 | 1.000 | 0.029 | 0.000 | 0.108 |
| LYC | 0.000 | 0.038 | 0.000 | 0.193 | 0.228 | 0.000 | 0.000 | 1.000 | 0.314 | 0.435 | 0.057 | 0.390 | 0.221 |
| LBH | 0.000 | 0.453 | 0.011 | 0.584 | 1.000 | - | 0.000 | 0.004 | 0.143 | 1.000 | 0.000 | 0.585 | 0.344 |
| JGC | 0.000 | 0.605 | 1.000 | 1.000 | 1.000 | - | 0.000 | 0.019 | 0.442 | 0.005 | 0.000 | 0.225 | 0.391 |
| DAL | 0.000 | 0.197 | 0.016 | 0.365 | 0.486 | 0.214 | 0.000 | 0.030 | 0.756 | 1.000 | 0.000 | 0.000 | 0.255 |
| GEM | 1.000 | 0.001 | 0.000 | 0.044 | 0.993 | 0.000 | 0.000 | 0.003 | 0.001 | 0.001 | 0.000 | 0.000 | 0.170 |
| HHY | 0.002 | 0.488 | 0.000 | 0.009 | 0.265 | - | 0.000 | 0.043 | 0.001 | 0.048 | 0.001 | 1.000 | 0.169 |
| NKK | 1.000 | 0.474 | 0.004 | 0.051 | 0.454 | 0.000 | 0.000 | 0.000 | 0.136 | 0.714 | 0.000 | 0.000 | 0.236 |
| GAZ | 0.170 | 0.236 | 0.041 | 0.025 | 0.909 | 0.016 | 0.000 | 0.172 | 0.025 | 0.026 | 0.000 | 0.000 | 0.135 |
| FTC | 0.000 | 0.021 | 0.002 | 0.000 | 0.011 | - | 0.000 | 0.026 | 0.003 | 1.000 | 0.030 | 1.000 | 0.190 |
| ZYY | 0.004 | 0.555 | 0.000 | 0.319 | 0.052 | - | 0.000 | 0.683 | 0.000 | 1.000 | 0.000 | 0.000 | 0.238 |
| CAJ | 0.000 | 0.000 | 0.000 | 0.011 | 0.002 | 0.000 | 0.000 | 0.000 | 0.002 | 1.000 | 0.000 | 0.000 | 0.085 |
| LGD | 0.000 | 0.000 | 0.001 | 0.000 | 0.000 | 0.000 | 0.000 | 0.000 | 0.000 | 0.000 | 0.000 | - | 0.000 |
| LHN | 0.000 | 0.000 | 0.000 | 0.000 | 0.000 | - | 0.000 | 0.000 | - | 0.000 | 0.000 | - | 0.000 |

**Additional file 2: Table S3.** *F_IS_* Per population

|  | MML | NAM | RJL | JGL | LYC | LBH | JGC | DAL | GEM | HHY | NKK | GAZ | FTC | ZYY | CAJ | LGD | LHN |
| --- | --- | --- | --- | --- | --- | --- | --- | --- | --- | --- | --- | --- | --- | --- | --- | --- | --- |
| AC10 | 0.456 | 0.19 | 0.397 | 0.214 | 0.168 | 0.835 | 0.499 | 0.42 | -0.129 | 0.382 | -0.07 | -0.018 | -0.011 | -0.252 | 0.658 | 0.476 | 0.6 |
| 1209 | -0.236 | -0.091 | -0.067 | 0.124 | 0.061 | -0.088 | -0.151 | 0.147 | 0.435 | -0.239 | -0.183 | -0.168 | -0.345 | -0.168 | 0.427 | -0.048 | 0.126 |
| AG3 | 0.307 | 0.162 | 0.347 | -0.362 | 0.608 | 0.243 | -0.053 | 0.219 | 0.666 | 0.025 | 0.104 | 0.231 | 0.174 | 0.418 | 0.499 | 0.209 | 0.031 |
| TTC5 | 0.257 | 0 | -0.047 | -0.554 | -0.168 | -0.213 | -0.089 | -0.028 | 0.344 | -0.11 | -0.106 | 0.085 | -0.252 | -0.118 | 0.258 | -0.036 | -0.028 |
| AAG6 | -0.096 | 0.001 | -0.012 | -0.236 | -0.172 | -0.073 | -0.026 | 0.024 | 0.01 | -0.116 | -0.108 | -0.152 | 0.369 | -0.372 | 0.241 | -0.047 | -0.497 |
| AAT1 | 0.591 | 0.729 | 0.279 | 0.859 | 0.686 | NA | NA | 0.102 | 0.814 | NA | 0.661 | 1 | NA | NA | 0.518 | 0.513 | NA |
| TTGT | 0.618 | 0.598 | 0.684 | 0.392 | 0.741 | 0.643 | 0.65 | 0.664 | 0.611 | 0.63 | 0.701 | 0.761 | 0.855 | 0.836 | 0.799 | 0.513 | 0.8 |
| AC1 | 0.795 | -0.03 | 0.659 | NA | -0.033 | 0.376 | 0.409 | -0.004 | 0.591 | 0.365 | 0.685 | 0.253 | 0.125 | -0.107 | 0.657 | 0.708 | 0.016 |
| CGA | 0.142 | -0.374 | -0.025 | 0.298 | -0.259 | -0.296 | 0.161 | 0.115 | 0.645 | -0.622 | 0.031 | -0.444 | -0.566 | 0.6 | 0.137 | -0.025 | NA |
| GAT | 0.401 | -0.079 | -0.033 | -0.031 | -0.165 | -0.019 | 0.787 | -0.082 | 0.469 | 0.659 | -0.1 | 0.083 | -0.127 | -0.033 | -0.034 | 0.438 | 0.512 |
| TC | 0.248 | 0.744 | 0.755 | 0.137 | 0.236 | 0.345 | 0.337 | 0.469 | 0.619 | 0.366 | 0.738 | 0.613 | 0.41 | 0.42 | 0.645 | 0.746 | 0.063 |
| AG5 | -0.01 | 0.279 | 0.169 | 0.797 | 0.113 | 0.009 | 0.043 | 0.442 | 0.92 | -0.054 | 0.574 | 0.94 | -0.054 | 0.605 | 0.806 | NA | NA |
| Mean | 0.226 | 0.182 | 0.247 | 0.094 | 0.168 | 0.228 | 0.253 | 0.231 | 0.486 | 0.106 | 0.212 | 0.204 | 0.071 | 0.212 | 0.481 | 0.296 | 0.209 |

**Additional file 2: Table S4**. Population *F_ST_* (lower left) and *N_m_* (top right) matrix table

| MML | NAM | RJL | JGL | LYC | LBH | JGC | DAL | GEM | HHY | NKK | GAZ | FTC | ZYY | CAJ | LGD | LHN |  |
| --- | --- | --- | --- | --- | --- | --- | --- | --- | --- | --- | --- | --- | --- | --- | --- | --- | --- |
|  | **14.339** | 8.359 | 4.168 | 4.481 | 2.933 | 2.165 | 3.968 | 4.537 | 2.790 | 1.443 | 2.649 | 1.929 | 2.060 | 3.381 | 1.394 | 1.242 | MML |
| **0.017** |  | 9.157 | 3.729 | 4.846 | 3.897 | 2.723 | 5.208 | 4.964 | 3.643 | 1.631 | 3.660 | 2.243 | 2.339 | 3.989 | 1.685 | 1.216 | NAM |
| 0.029 | 0.027 |  | 5.343 | 4.631 | 3.591 | 3.007 | 3.244 | 3.824 | 2.664 | 1.256 | 2.471 | 1.839 | 2.150 | 3.110 | 1.506 | 1.656 | RJL |
| 0.057 | 0.063 | 0.045 |  | 2.379 | 2.165 | 1.980 | 2.054 | 2.619 | 1.392 | 0.857 | 1.796 | 1.143 | 1.320 | 2.292 | 1.150 | 1.601 | JGL |
| 0.053 | 0.049 | 0.051 | 0.095 |  | 1.915 | 1.796 | 3.241 | 3.635 | 3.291 | 1.788 | 2.337 | 2.466 | 2.210 | 2.928 | 1.375 | 1.236 | LYC |
| 0.079 | 0.060 | 0.065 | 0.104 | 0.115 |  | 11.771 | 2.764 | 2.931 | 2.657 | 1.105 | 3.506 | 1.863 | 2.456 | 3.187 | 1.960 | 1.458 | LBH |
| 0.104 | 0.084 | 0.077 | 0.112 | 0.122 | 0.021 |  | 2.410 | 2.731 | 2.121 | 0.977 | 2.729 | 1.718 | 2.294 | 2.820 | 1.567 | 1.570 | JGC |
| 0.059 | 0.046 | 0.072 | 0.109 | 0.072 | 0.083 | 0.094 |  | 4.617 | 6.371 | 2.889 | 8.296 | 5.165 | 5.391 | 7.763 | 1.780 | 1.425 | DAL |
| 0.052 | 0.048 | 0.061 | 0.087 | 0.064 | 0.079 | 0.084 | 0.051 |  | 2.439 | 1.627 | 3.012 | 2.179 | 2.166 | 4.861 | 1.359 | 1.516 | GEM |
| 0.082 | 0.064 | 0.086 | 0.152 | 0.071 | 0.086 | 0.105 | 0.038 | 0.093 |  | 2.911 | 4.838 | 10.767 | 7.846 | 3.260 | 1.443 | 0.952 | HHY |
| 0.148 | 0.133 | 0.166 | 0.226 | 0.123 | 0.184 | 0.204 | 0.080 | 0.133 | 0.079 |  | 2.861 | 3.199 | 2.748 | 2.592 | 1.257 | **0.807** | NKK |
| 0.086 | 0.064 | 0.092 | 0.122 | 0.097 | 0.067 | 0.084 | 0.029 | 0.077 | 0.049 | 0.080 |  | 3.789 | 5.376 | 8.027 | 3.052 | 1.544 | GAZ |
| 0.115 | 0.100 | 0.120 | 0.180 | 0.092 | 0.118 | 0.127 | 0.046 | 0.103 | 0.023 | 0.072 | 0.062 |  | 8.537 | 2.967 | 1.252 | 0.902 | FTC |
| 0.108 | 0.097 | 0.104 | 0.159 | 0.102 | 0.092 | 0.098 | 0.044 | 0.103 | 0.031 | 0.083 | 0.044 | 0.028 |  | 4.227 | 1.547 | 1.123 | ZYY |
| 0.069 | 0.059 | 0.074 | 0.098 | 0.079 | 0.073 | 0.081 | 0.031 | 0.049 | 0.071 | 0.088 | 0.030 | 0.078 | 0.056 |  | 3.235 | 1.733 | CAJ |
| 0.152 | 0.129 | 0.142 | 0.179 | 0.154 | 0.113 | 0.138 | 0.123 | 0.155 | 0.148 | 0.166 | 0.076 | 0.166 | 0.139 | 0.072 |  | 1.329 | LGD |
| 0.168 | 0.171 | 0.131 | 0.135 | 0.168 | 0.146 | 0.137 | 0.149 | 0.142 | 0.208 | **0.236** | 0.139 | 0.217 | 0.182 | 0.126 | 0.158 |  | LHN |
